# The Effects of Sampling Location and Predictor Point Estimate Certainty on Posterior Support in Bayesian Phylogeographic Generalized Linear Models

**DOI:** 10.1038/s41598-018-24264-8

**Published:** 2018-04-12

**Authors:** Daniel Magee, Jesse E. Taylor, Matthew Scotch

**Affiliations:** 1 Department of Biomedical Informatics, Arizona State University, Tempe, Arizona USA; 2 Biodesign Center for Environmental Health Engineering, Arizona State University, Tempe, Arizona USA; 3 School of Mathematical and Statistical Sciences, Arizona State University, Tempe, Arizona USA; 4 School of Life Sciences, Arizona State University, Tempe, Arizona USA

## Abstract

The use of generalized linear models in Bayesian phylogeography has enabled researchers to simultaneously reconstruct the spatiotemporal history of a virus and quantify the contribution of predictor variables to that process. However, little is known about the sensitivity of this method to the choice of the discrete state partition. Here we investigate this question by analyzing a data set containing 299 sequences of the West Nile virus envelope gene sampled in the United States and fifteen predictors aggregated at four spatial levels. We demonstrate that although the topology of the viral phylogenies was consistent across analyses, support for the predictors depended on the level of aggregation. In particular, we found that the variance of the predictor support metrics was minimized at the most precise level for several predictors and maximized at more sparse levels of aggregation. These results suggest that caution should be taken when partitioning a region into discrete locations to ensure that interpretable, reproducible posterior estimates are obtained. These results also demonstrate why researchers should use the most precise discrete states possible to minimize the posterior variance in such estimates and reveal what truly drives the diffusion of viruses.

## Introduction

Ancestral state reconstruction has long been an important topic in phylogenetic research^1^. Recent years have seen a turn to a Bayesian statistical framework to estimate posterior support of ancestral states^2^, making use of a Bayesian stochastic search variable selection (BSSVS) procedure^3,4^. Although this popular hypothesis-testing framework is effective in identifying root locations with high probability, the posterior probability of ancestral states is drawn exclusively from genomic features. While this interpretation certainly holds value in identifying evolutionary relationships, it does not allow for a direct characterization of the effects of suspected epidemiological factors on evolution and diffusion.

In addition to the lack of external influence on the phylogenies, studies in a discrete Bayesian phylogeographic setting must account for the nontrivial issue of identifying geographic sampling locations and, on occasion, pooling multiple locations into a single discrete state or splitting a continuous geographic area or administrative region into multiple states. A straightforward approach is to combine adjacent administrative divisions (*e.g*. neighboring countries) which are often divided by arbitrary boundaries, such as a parallel latitude, mountain range, or river, but these combinations may lose the value that each location holds individually. Specifically, population demographics, cultural aspects, and medical and agricultural practices may widely differ in adjacent locations. Furthermore, these features may vary in specific areas of each individual location, so these differences should be accounted for in some capacity. Failure to do so may lead to biased posterior probability estimates along any branch in the phylogeny. Pooling has been used in multiple phylogeographic studies, including countries and air communities^5^, islands in the Caribbean^6^, and states in the U.S.^7^, while splitting of countries or other administrative regions into multiple states is also commonly performed^2,5,8^.

The development and implementation of a generalized linear model (GLM) in Bayesian phylogeography has enabled the modeling of transition rate matrices as a function of biologically relevant predictors^5^. This framework was first used to evaluate the global diffusion of H3N2 influenza^5^ and has subsequently implemented to assess additional pathogens, such as H5N1 influenza in Egypt^8^ and HIV in Brazil^9^, among others. These studies are able to accommodate properties of the discrete states themselves, such as demographic, environmental, and geographic features. Thus, the framework models the spread of the pathogen as a joint likelihood of the genetic data and the predictor data. Posterior inclusion probability estimates are available for each predictor, and Bayes factors (BFs) can be used to evaluate the support for each predictor’s role in the spatiotemporal dynamics of the pathogen. Regression coefficients are also available for each predictor such that its contribution to the overall diffusion process can be quantified. Although these implementations of the phylogeographic GLM may provide advantages in biological interpretation of phylogenies and identify driving forces behind widespread diffusion, the issue of predictor aggregation remains. Namely, each of these studies used point estimates of their predictors at high levels of spatial order, such as continent or country-wide averages, often due to a lack of more specific sampling locations or the inability to assign predictor data to a more local level. While these estimates are not inherently inaccurate, the variance of a temperature predictor, for example, may be rather large when considering the local differences in climate across such a large area. This calls into question whether a point estimate of a predictor over a large geographic area will enable accurate estimates of posterior predictor support.

In this study, we investigate the effects of aggregating predictor data for phylogeographic GLMs at different spatial scales. Specifically, we examine how changes in the accuracy of the predictor point estimates may alter their respective posterior inclusion probabilities and regression coefficients. For example, climate is known to contribute to the global source-sink dynamic of influenza viruses^10^, but temperature and precipitation are certainly not constant throughout the regions used as discrete states in many cases. Here, we hypothesize that as point estimates of the predictors become more representative of the geographic sampling location, posterior variance of the supported predictors will be minimized. This reduction in variance should provide more confidence in ensuing biological interpretations of the pathogen-predictor relationship.

We use West Nile virus (WNV) in the U.S. as a case study to address this question and gain insight for researchers that wish to utilize the GLM framework. WNV is a vector-borne virus that first emerged in the U.S. in 1999^11,12^ and has resulted in over 41,000 human infections in the country^13^. These infections occur primarily through bites of infected mosquitos of the *Culex* genus^14^, although many bird species are natural hosts^15^. To our knowledge, there has been no prior study on WNV that has utilized a phylogeographic GLM. Here, we discretized 299 sequences of WNV by U.S. Census Bureau (USCB) regions (CBR), USCB subdivisions (CBS), state, and county of isolation and perform a separate aggregation of predictor data at each level. We additionally perform an assessment at the county level for each of the four CBRs. This study will critically evaluate the impact of discretization of predictor data on a phylogeographic GLM, providing researchers with empirical evidence of how variables contributing to the diffusion of viruses can change given differences in discrete state partitioning and the level at which accurate point estimates of predictor values can be obtained.

## Results

### Predictor Correlations

At the highest level of aggregation, CBR, the GLM’s predictor matrix was not of full rank, which is required to run GLM analyses in BEAST. In fact, of the 105 pairwise predictor-predictor combinations, six show a very strong linear correlation at the CBR level, which is the total number of such instances in the remaining seven models. We list highly-correlated predictors (|Pearsons’ r| >0.9) for all models in Table 1. From Table 1, 12 of the 15 predictors showed a high correlation with another predictor in at least one model, with only *Corvidae* average counts at the location of origin, distance, and unvaccinated horses at the location of destination failing to do so. Although the CBS, Midwest, and South models did exhibit some strong correlations between predictors (Table 1), each predictor matrix achieved full rank.

**Table 1:** Predictor combinations where |Pearson’s r| >0.9.

Model	Predictor 1^a^	Direction 1	Predictor 2^a^	Direction 2	Pearson’s r
CBR	CA	Destination	PC	Destination	−0.90
CBR	CC	Destination	PD	Destination	−0.93
CBR	CC	Origin	PD	Origin	−0.95
CBR	PC	Destination	WL	Destination	>0.99
CBR	PC	Origin	WL	Origin	>0.99
CBR	TP	Destination	UH	Destination	>0.99
CBS	PC	Origin	TP	Origin	0.95
CBS	PC	Destination	WL	Destination	0.95
Midwest	TP	Destination	TP	Origin	0.96
South	TP	Destination	TP	Origin	0.99
South	CC	Origin	PD	Origin	0.92
South	CC	Destination	PD	Destination	0.91

^a^(CA) *Corvidae* counts; (CC) case counts; (PC) precipitation; (PD) population density; (TP) temperature; (UH) unvaccinated horses; (WL) wetlands.

### MCC Metrics and Population Demographics

For the CBS, state, and national county aggregations, each MCC phylogeny exhibits similar posterior statistics, which we summarize in Table 2. Specifically, the time to the most recent common ancestor (tMRCA) and its highest posterior density (HPD) places the root of the viral tree in the late 1990s while the location is identified in the Northeastern U.S. Root states for the national models show “New England”, “Connecticut”, and “Fairfield County, Connecticut” for the CBS, state, and national county aggregations, respectively. The root state posterior probability (RSPP) is highest for the state aggregation (p = 0.98) followed by the CBS and county aggregations (p = 0.94 and 0.86, respectively). The Kullback-Leibler (KL) divergence increases from the CBS to state to county aggregation in the three national models. For the Midwest, South, and West regional county analyses, each molecular clock rate’s HPD range is larger than any of the national analyses. The oldest sampling dates for the Midwest, Northeast, South, and West counties were 2002, 1999, 2001, and 2003, respectively, and the tMRCAs of the viral samples from these four regional models were estimated to be 2000, 1997, 1998, and 2001, respectively. The RSPPs of the South and West models (p = 0.63 and 0.54, respectively) are substantially lower than those from the Midwest and Northeast models (p = 0.99 and 0.95, respectively), and the South model achieves the weakest KL divergence (1.52) of all models. We note a strong linear correlation between the number of discrete states and KL divergence for all seven models (Pearson’s r = 0.99), and also between the percent identical sites and number of taxa per model (Pearson’s r = −0.99). We have uploaded the MCC phylogeny for each model to FigShare (https://figshare.com/projects/WNV_GLM_Aggregation_Study/19201).

**Table 2:** Posterior statistics of the MCC phylogenies.

Model^a^	Identical Sites (%)	Clock Rate (95% HPD)	tMRCA (95% HPD)	Root Location	RSPP	KL
CBS	79.2	7.4 × 10^−4^ (6.1–8.7 × 10^−4^)	1997.5 (1995.9–1998.5)	New England	0.94	3.48
State	79.2	7.2 × 10^−4^ (5.9–8.6 × 10^−4^)	1997.6 (1996.1–1998.6)	Connecticut	0.98	48.27
County	79.2	6.8 × 10^−4^ (5.7–7.9 × 10^−4^)	1997.5 (1996.1–1998.6)	Fairfield County, CT	0.86	233.18
Midwest	96.5	4.3 × 10^−4^ (1.7–7.2 × 10^−4^)	2000.3 (1997.3–2001.4)	Cook County, IL	0.99	47.70
Northeast	87.9	6.7 × 10^−4^ (5.2–8.3 × 10^−4^)	1997.7 (1996.4–1998.7)	Fairfield County, CT	0.95	85.67
South	91.5	8.1 × 10^−4^ (3.2–12.1 × 10^−4^)	1998.8 (1996.0–2000.7)	Harris County, TX	0.63	1.52
West	95.8	5.3 (2.0–8.6 × 10^−4^)	2001.6 (1999.0–2002.7)	Park County, CO	0.54	17.25

^a^Results not available for the CBR phylogeny as its predictor design matrix did not achieve full rank.

The three national models demonstrate similar trends in population demographics via Bayesian Skyline plots as well. The genetic diversity shows a sharper decline in the county model than the CBS or state models near the year 2003, but the remainder of the Skylines are nearly identical among the three models. We provide the Bayesian Skyline plots for the national and regional models on FigShare (https://figshare.com/projects/WNV_GLM_Aggregation_Study/19201). The Skyline plot of the Northeast county-level model appears similar to that of the three national models over its time frame, which is likely an artifact of the density of samples in the Northeast region compared to the other three regions. The Midwest, South, and West models show generally steady levels of diversity across their respective time periods. While the phylogenies for the CBS, state, and national county models show generally consistent results, this is not true of the predictors included in these three models.

### Predictor Support Metrics

**Figure 1:**
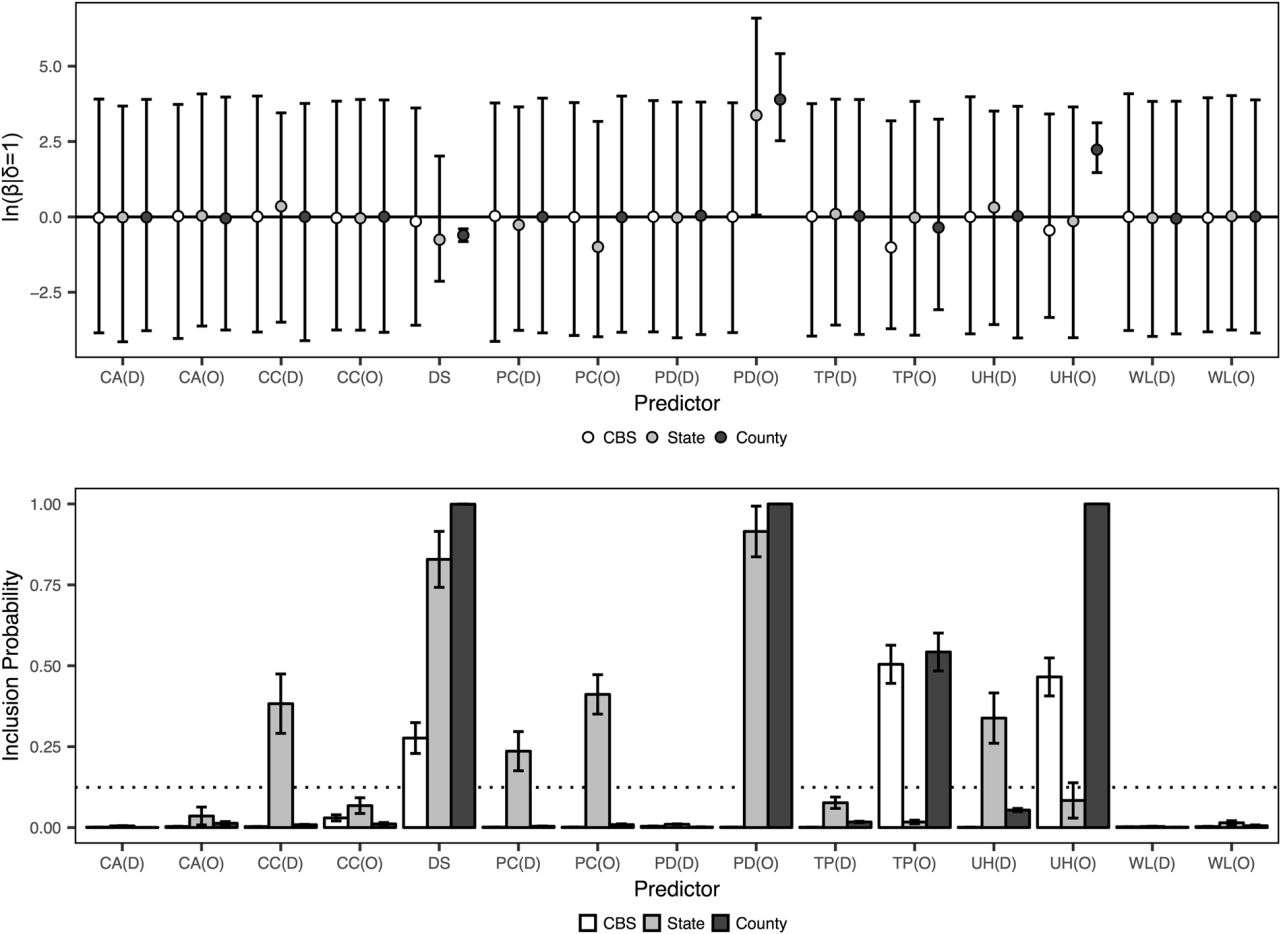
Posterior metrics for the predictors in the three national models. Inclusion probabilities for the predictor indicators (δ) and corresponding regression coefficients (β) for the 15 predictors for the CBS, state, and county aggregations. The dotted line corresponds to BF = 3. Error bars represent the standard error for each predictor’s inclusion probability and the 95% HPD for each predictor’s regression coefficient. Predictors are annotated with their evaluation from location of origin (O) and location of destination (D).

We show the posterior inclusion probabilities and corresponding regression coefficients for the CBS, state, and county aggregations in Fig. 1. Here, the *Corvidae* counts and wetlands predictors fail to achieve a BF > 3 from either location of origin or location of destination in any of the three aggregations. For case counts and precipitation, the CBS and county models yield BF < 3 from both location of origin and location of destination, while the state model achieves BF support for case counts at the location of destination and precipitation at both the location of origin and destination (BF = 13.1, 14.8, and 6.5, respectively). The distance predictor shows the most scale-dependent behavior, as support increases from the CBS to the state to the county levels (BF = 8.1, 102.4, and 30,185.0, respectively). Furthermore, the 95% HPD of the regression coefficient of the distance predictor decreases at each level of aggregation (95% HPD range = 7.21, 4.15, and 0.42 for the CBS, state, and county aggregations, respectively, in log-space). The entire HPD is negative for the county aggregation, which suggests that distance is preventing the diffusion of WNV in that model. The trend of decreasing posterior variance of the regression coefficient also holds true for population density at the location of origin (95% HPD range = 7.62, 6.53, and 2.89 for the CBS, state, and county aggregations, respectively, in log-space). Here, the entire HPD is positive for this predictor at the state and county aggregations, which suggests that population density at the location of origin is driving the diffusion of WNV in these two models. The CBS aggregation yields BF = 0.03 for this predictor, while the state aggregation yields BF = 227.6. For the county aggregation, this predictor was included in every sample after the 10% burn-in period, which corresponds to an inclusion probability of 1.0 and a Bayes factor that tends to infinity. This is also true for the county aggregation of the unvaccinated horses data at the location of origin, and the entire 95% HPD of the regression coefficient is positive, indicating that this predictor is also driving the diffusion of WNV for the county model. The CBS aggregation shows support for this predictor while the state aggregation does not (BF = 18.4 and 1.9, respectively). The state aggregation does show support for unvaccinated horses at the location of destination (BF = 10.8) while the CBS and county aggregations do not (BF = 0.02 and 1.20, respectively). Finally, the CBS and county aggregations show similar support for temperature at the location of origin (BF = 21.5 and 25.1, respectively), while the state aggregation does not (BF = 0.4).

**Figure 2:**
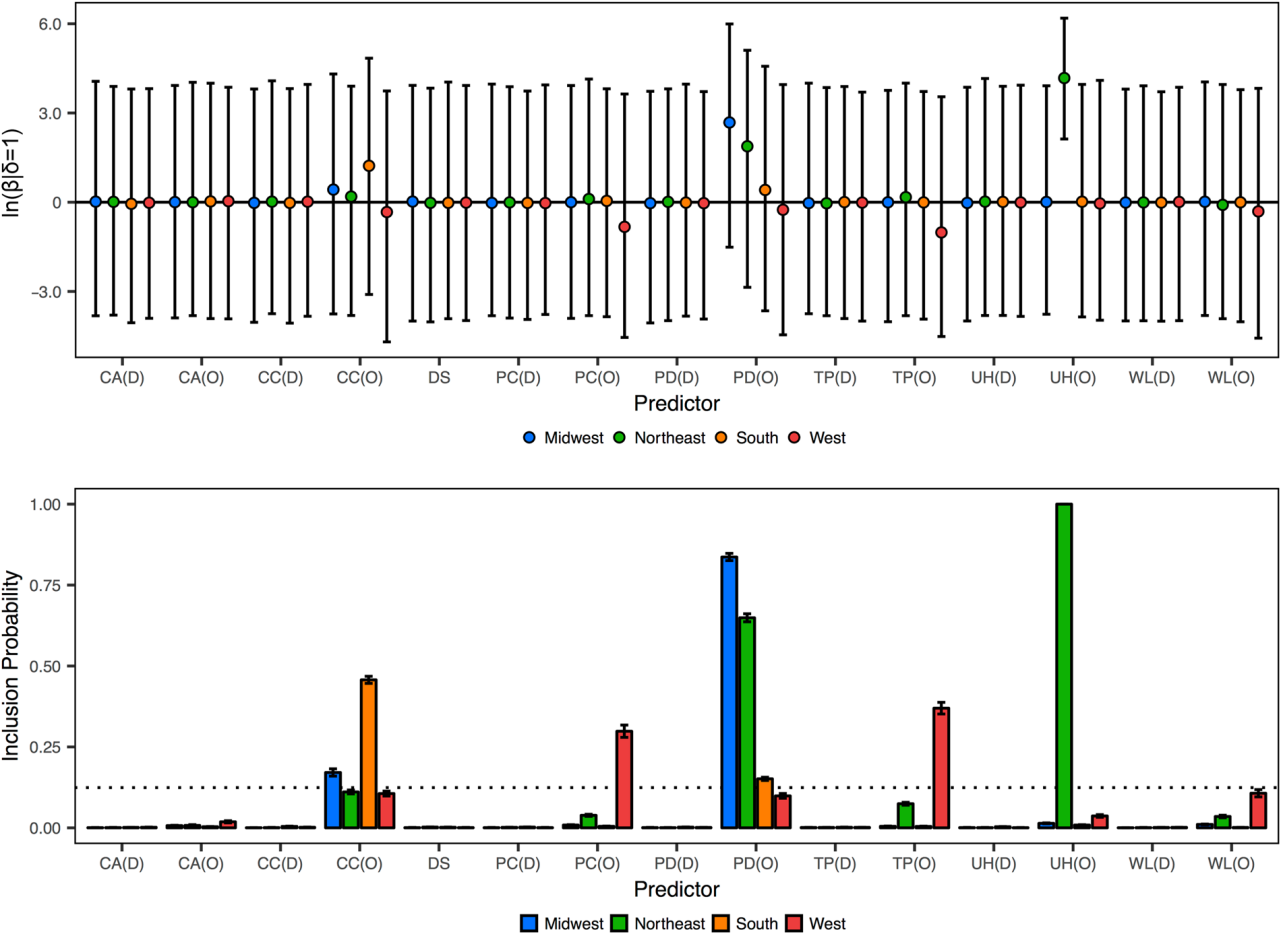
Posterior metrics for the predictors in the four county-level regional models. Inclusion probabilities for the predictor indicators (δ) and corresponding regression coefficients (β) for the 15 predictors for Midwest, Northeast, South, and West models. The dotted line corresponds to BF = 3. Error bars represent the standard error for each predictor’s inclusion probability and the 95% HPD for each predictor’s regression coefficient. Predictors are annotated with their evaluation from location of origin (O) and location of destination (D).

We also show the posterior predictor data for the four regional models at the county level in Fig. 2. Here, we again see that the *Corvidae* counts and wetlands area predictors fail to achieve BF support from either location of origin or location of destination in any of the four regional models. Of the 15 predictors included, only case counts, precipitation, population density, temperature, and unvaccinated horses, each from the location of origin, showed BF > 3 in these models. Only case counts and population density were supported in more than one of the regional models. For the Northeast model, unvaccinated horses at the location of origin yields a positive 95% HPD, which suggests that this predictor was also driving viral propagation in this region. This is consistent with the national county aggregation (Fig. 1). The BF for this predictor tends to infinity in the Northeast model, and this model also shows support for population density at the location of origin (BF = 39.1). In the Midwest model, case counts and population density are supported (BF = 4.4 and 108.3, respectively). In the South model, case counts and population density at the location of origin are supported (BF = 17.8 and 3.8, respectively). The West model only shows support for temperature and precipitation at the location of origin (BF = 12.4 and 9.0, respectively).

**Figure 3:**
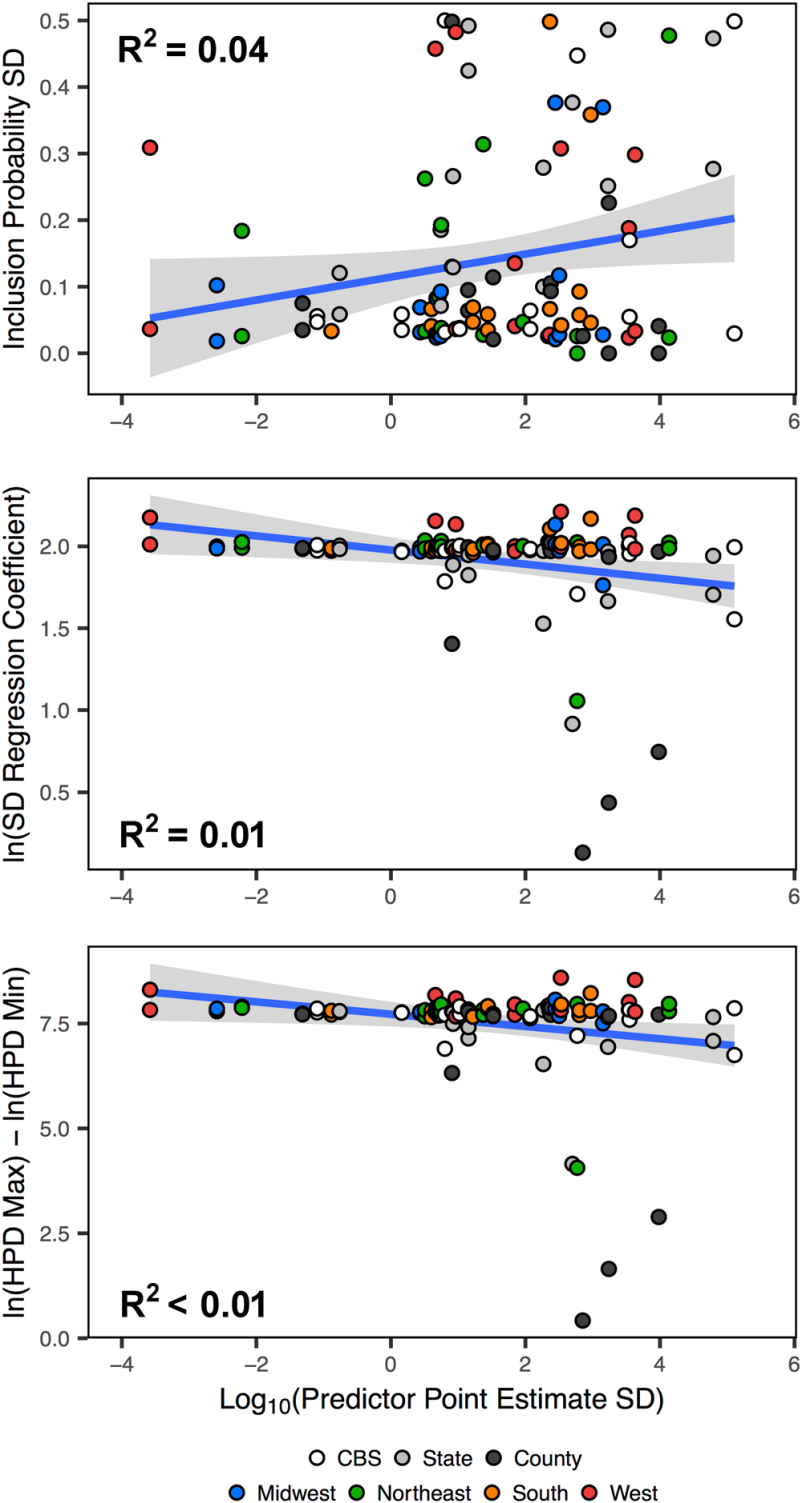
Linear correlations between the variance of predictor point estimates and the variance in posterior support metrics. The blue lines represent the lines of best fit and the shaded areas represent the 95% confidence intervals, and include data for all national and regional models.

While Fig. 1 outlines the variance in predictor support given the level of spatial aggregation and Fig. 2 shows that local predictor trends are not necessarily consistent with those observed on a national basis, it is also pertinent to analyze possible sources of variance in the posterior estimates. In Fig. 3, we plot the variance of the inclusion probabilities and corresponding regression coefficients against the variance of the predictor point estimates for each individual model. That is, we show the posterior estimates as a function of the known variance in predictor point estimates. From Fig. 3, the 95% confidence intervals fail to encapsulate many of the data points for any of the three statistics. The low R^2^ values indicate that the variance in posterior estimates are not linearly correlated with the variance in predictor point estimates.

### Consistency of Predictor Support

**Figure 4:**
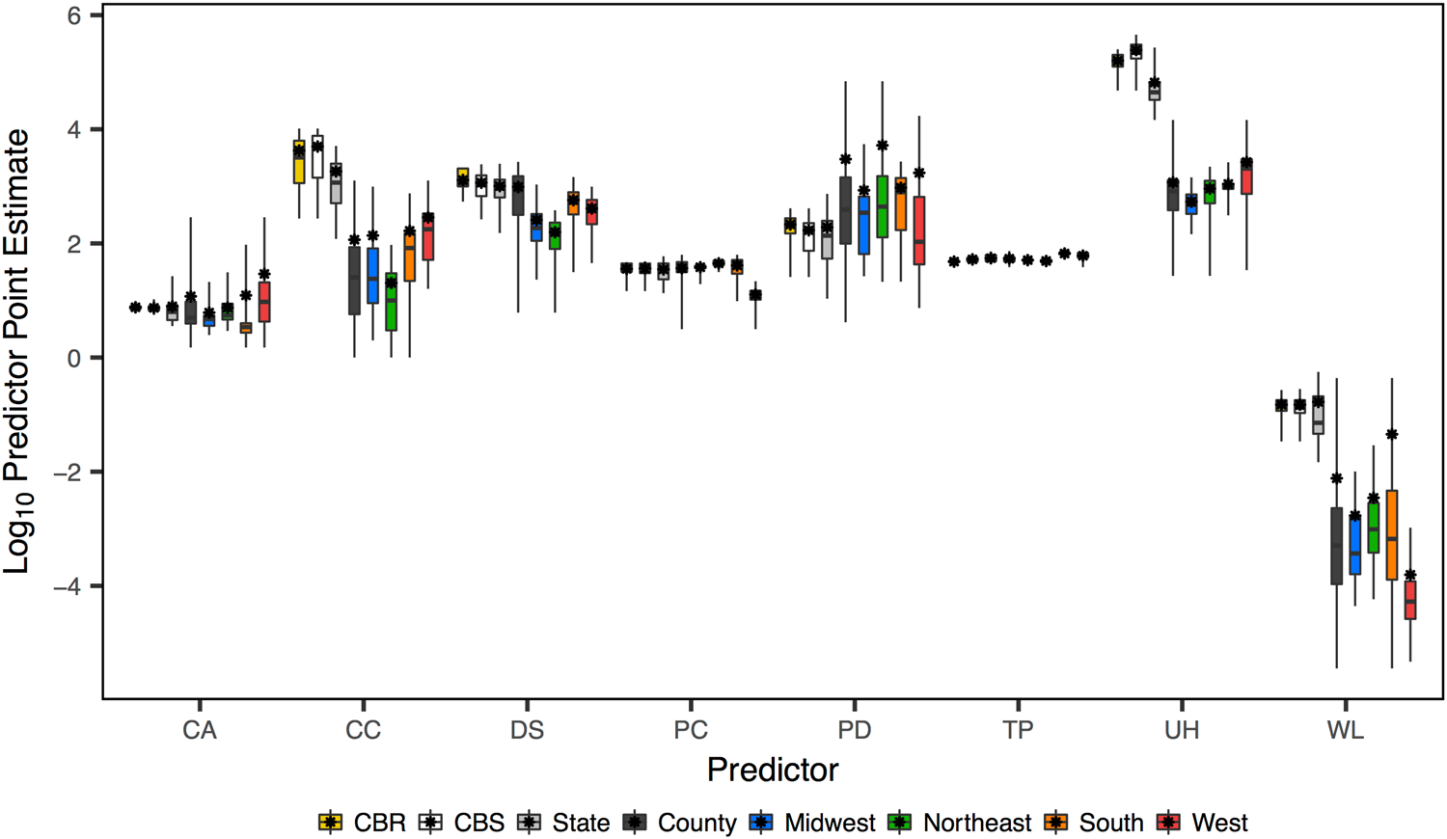
Predictor point estimate distributions for each model. Predictor abbreviations are: *Corvidae* average counts (CA), case counts (CC), distance (DS), precipitation (PC), population density (PD), temperature (TP), expected unvaccinated horses (UH), and wetlands area (WL).

We find the distance predictor to be of particular interest, especially pertaining to the three national models. Here, there is an increase in predictor support as the knowledge of the sampling location goes from most uncertain (CBS, BF = 8.1) to moderately uncertain (state, BF = 102.4) to least uncertain (county, BF = 30,185.0). Furthermore, the range of the HPD decreases as the sampling location certainty increases. The county-level aggregation suggests that distance is limiting the spread of WNV in the U.S., as its entire HPD is negative. The geographic diffusion of WNV in the U.S. is known to have occurred rapidly^16^. Thus, as the distribution of pairwise distances among discrete locations is largest at the county-level (Fig. 4), it is plausible that the distance predictor would be protective. We note that the distance predictor is not supported in any of the four regional county-level models, which could indicate that geographic distance is less important at the local level but more important for widespread diffusion dynamics. Alternatively, this could simply be a result of the fewer sequences and less genetic diversity available in the local analyses compared with the national analyses (Table 2). The strong negative correlation (Pearson’s r = −0.99) between the number of viral sequences and the percent of identical sites per model demonstrates that an increase in the number of sequences results in a decrease in the number of fixed sites, and thus an increase in genetic diversity for the national models.

In addition to the distance predictor, human population density at the location of origin is not supported at the CBS level (BF < 0.1), well-supported at the state level (BF = 227.6), and was found to be included in every sample for the national county model (BF tends to infinity). This predictor is also supported in the Midwest, Northeast, and South regional county models (BF = 108.3, 39.1, and 3.8, respectively), but not for the West model (BF = 2.3). The point estimates of this predictor are the least uncertain at the county-level, so its unanimous support in the national model and frequent support in the regional county-level models provide evidence that population density is likely involved in WNV diffusion. As this predictor’s contribution is strictly positive (regression coefficient = 3.9 and 95% HPD = [2.5, 5.4] in log-space), we would conclude that it is driving the diffusion of WNV from county-to-county at the national level, however its failure to be supported in more sparse aggregations gives us pause.

As the remaining predictors have variable support among the CBS, state, and county-level analyses, we reiterate several points about the point accurate estimations of two predictors: human case counts and expected unvaccinated horses. For the human case counts, we collected the data at the state-level^13^ and not the specific county. Thus, our CBS and state aggregations have an accurate point estimate for this predictor, but for the county aggregation we assumed that the case counts per county were proportional to the county’s population within the state. This assumption is not necessarily correct for the county-level aggregations. Case counts at the location of destination is supported in only the state model (BF = 13.1) so, the assumption for the county aggregations does not appear to have resulted in a potentially misleading supported predictor, although it is unknown how an alternative estimation assumption would change the posterior results. The expected unvaccinated horses predictor, however, does result in potentially suspect support metrics. The population of horses is known at the state-level^17^, and thus the population per CBS is simply the sum of the states in the region. For the county-level aggregation, we assumed that the number of horses was uniformly distributed across the state and thus that the number of horses per county was proportional to its land area. This predictor also required the vaccination rate per state, and several states in our dataset (Arizona, Connecticut, Ohio, Nebraska, South Dakota, and Texas) were absent from the survey from which we obtained this predictor^18^. We note that at least one state is absent from the Midwest, Northeast, South, and West regions of the USCB, which directly impacts the regional county-level analyses as well. We assumed that the absent states had the same vaccination rate as the most proximal geographic region from that survey, and that the CBS estimates were the average of the states in the region. We also assumed that the vaccination rate in each county was the same as the vaccination rate per state, which creates additional uncertainty. Overall, each of the seven completed models required a certain degree of assumption and potential error introduction for this predictor, but the county-level aggregations required one additional assumption, thus increasing its uncertainty. We found that the expected unvaccinated horses at the region of origin is facilitating the diffusion of WNV in both the national and Northeast county-level aggregations (Figs 1 and 2) (BF tends to infinity and 95% HPD of the regression coefficient is strictly positive for each) and is supported at the CBS-level (BF = 18.4) as well (Fig. 1), although its directionality is uncertain. Because the state-level point estimate is likely the most accurate for the expected unvaccinated horses predictor, and as we found that this predictor is supported from the location of destination in that model (BF = 10.8) we question the findings of the CBS and county-level aggregations. It is likely that multicollinearity is influencing the support of this predictor at the county level. For the CBS, state, and national county-level aggregations, the correlation between our point estimate for expected unvaccinated horses and the size of the discrete state is 0.23, 0.66, and 0.86, respectively. For the Midwest, Northeast, South, and West regional county-level models, the correlations are 0.62, 0.73, 0.67, 0.89, respectively. These data indicate that any support for the unvaccinated horses predictor in any of the county-level aggregations is rather indicative of the size of the discrete states, not the horse population, and should further caution researchers when aggregating predictors where assumptions must be made. In addition, horses infected with WNV are not known to be capable of passing the virus back to uninfected mosquitos, nor can they infect other horses or humans^19–21^, so the suggestion that unvaccinated horses contributing to the spread of WNV seems suspect. Further details on these predictors can be found in *Materials and Methods*.

We list the R^2^ value for linear models between the predictor point estimate accuracy (independent variable) and posterior statistic variance (dependent variable) for each individual analysis in Table 3. From Table 3, 20% of the posterior variance of the regression coefficient is explained by the predictor point estimate variance in the CBS-level aggregation, although just 8% of the posterior variances of the inclusion probability and HPD range of the regression coefficient are explained by the predictor point estimate variance. The state and national county aggregations do not yield R^2^ > 9% for any of the three statistics. For the regional county-level models, a modest amount of the variance in posterior estimates are explained by the predictor point estimate variances in the Midwest, Northeast, and South models. The Midwest analysis yields R^2^ > 17% for each linear model, and 24% of the variance in the posterior regression coefficient is explained. Meanwhile, the West analysis shows R^2^ ≤ 1% for all three linear models.

**Table 3:** R^2^ statistics for linear models between the variance of predictor point estimates and the variance in posterior support metrics.

Model	Dependent Variable		
	SD P(δ^a^ = 1)	SD (β^b^)	HPD Range (β^b^)
CBS	0.08	0.20	0.08
State	0.09	<0.01	0.09
County	0.07	0.07	0.07
Midwest	0.17	0.24	0.17
Northeast	0.14	0.01	0.14
South	0.10	0.18	0.10
West	0.01	<0.01	0.01
Overall	0.04	0.01	<0.01

^a^Inclusion probability.

^b^Regression coefficient.

## Discussion

In this paper, we analyzed WNV using multiple Bayesian phylogeographic GLMs and compare posterior phylogeographic reconstructions and support for our included predictors. We hypothesized that as the uncertainty of the sampling location decreased, the posterior support variance would in turn decrease to give additional confidence to the predictors that were heavily involved in the spatiotemporal diffusion of the virus. Our data shows that this hypothesis is only true for the distance predictor among the three national models. For the remaining 14 predictors, the posterior support metrics were highly inconsistent, undermining our ability to confidently identify which predictors drove the spread of WNV across the U.S. We found that the MCC topology and the age of its root were similar in the three national models that were successfully executed (CBS, state, and county-level aggregations). The tMRCA for these viruses in each model is consistent with those presented in previous phylogeographic studies of WNV in the U.S.^11,22^. The observed molecular clock rates for each are slightly slower than the reported 5.06 × 10^−4^ in the open reading frame of human-origin isolates between 1999–2011 in the U.S.^22^, but we note that our study includes one additional year and accounts for all sampled mosquito and avian species as well. Thus, we conclude that the topology of the viral phylogenies is mainly determined by the sequence data rather than by the predictor data or discrete state partitioning. This is the first assessment of WNV that implements the GLM framework.

Although the correlations between the posterior predictor variance and variance in predictor point estimates fail to show linear trends, we do not consider this finding problematic. As the phylogeographic reconstructions are informed via both predictor data and sequence data^5^, identifying strong correlations would perhaps demonstrate a systematic bias within the GLM framework. Instead, these data may show the inherent stochasticity of this framework. Our inability to execute the CBR model due to its strong correlations between predictors indicates that discretizing locations and aggregating predictor data at highly uncertain levels for phylogeographic GLMs may require the elimination of predictors from the model and/or selection of alternate predictors such that the correlations are reduced. Either could result in a loss of pertinent information or misleading results regarding the dynamics of the virus in question.

Limitations of this study include our inability to replicate phylogeographic reconstructions of WNV in the U.S. using whole genome sequences and the lack of precise predictor data, in some cases, at the county level. The former reduces our ability to firmly conclude that our posterior predictor signals are a result of known WNV diffusions patterns, while the latter is a problem that researchers are likely to face with respect to the predictors that they desire to include. On that note, we believe that this study illustrates decisions that may arise when performing such studies and, consequentially, why alternative discretization should be considered prior to concluding results. Such considerations are especially important when an effort is made to ascertain implications for public health. While previous work^5^ has fixed a number of taxa and displayed posterior data for multiple spatial aggregations, our work is the first to demonstrate and emphasize the disparities in posterior metrics that may be observed with such methods.

Although researchers that employ a GLM in Bayesian phylogeography may be tempted to create inferences based on posterior support of predictors and subsequently provide biological justification of these findings, we believe that our results tell a cautionary story of the need to consider alternative discrete state construction. Here, we have shown how assigning identical nucleotide sequences into different discrete state sets with different degrees of spatial resolution can influence posterior support for predictors. As we are unable to pinpoint the source of the posterior variance as it pertains to the variance in point estimates across the discrete locations, we refrain from making any firm statements regarding which predictors are involved in the diffusion of WNV. Furthermore, as posterior predictor estimates obtained from regional county-level models are often inconsistent with those from the national level, it may also be important to perform additional analyses at the local level prior to stating conclusions regarding more widespread epidemics (and vice versa). Future work that utilizes a simulated phylogeny with known relationships to both the taxa and predictors will be necessary to investigate whether the there is some optimal spatial aggregation that exists for a given dataset. In addition, it is unclear if the variation in spatiotemporal sampling or in predictors are more likely to bias real-world investigations like in this study.

Finally, it is often the case that sampling locations are only known or annotated to low-level, uninformative, and ambiguous locations^23,24^. As we have shown here for the distance, population density, and expected unvaccinated horses predictors, aggregations that are averaged over a wider geographic area may not fully encapsulate or represent the true data at the precise sampling location, even though these same predictors at the county-level received strong support. Simply put, knowing the precise sampling location may enable local dynamics in viral diffusion to be revealed via GLM analyses, whereas this information may be lost when sequences are aggregated into coarser geographical units. Thus, we urge researchers that annotate and submit nucleotide sequences to public repositories to use the most precise sampling location possible so that these data can be used to accurately determine the factors that drive the diffusion of deadly viruses.

## Materials and Methods

### Model Parameters

#### Nucleotide Sequences

We obtained whole genome WNV sequences from the Virus Pathogen Database and Analysis Resource^25^ using the following search criteria: Family = *Flaviviridae*, Genus = *Flavivirus*, Species = *West Nile Virus*, Collection Year = *1999–2012*, Geography = *USA*, Host = *All*. This resulted in 781 sequences, 299 of which were annotated with a state of origin and county of origin. We aligned these 299 sequences using MAFFT v7.017 in Geneious Pro v.6.1.8 (Biomatters Ltd., Auckland, New Zealand). After exploratory Bayesian phylogeographic GLMs with our sequence set failed to replicate molecular clock rates observed for WNV in the U.S. over a similar time period^11,22,26^, we elected to focus on the envelope (E) protein of the WNV genome. We extracted the E protein for each record and aligned the sequences with the same parameters described above. We also performed four additional alignments, one for the sequences collected from each of the four CBRs: Midwest, Northeast, South, and West. We classified the hosts of these viruses using four categories: mosquito (n = 138), Corvidae (108), human (44), and other avian (9). The mosquito group contains members of the *Aedes* (11)*, Culex* (101)*, Culiseta* (15)*, Ochlertotatus* (9)*, and Psorophora* (2) genera. *Corvidae* is a family of birds, of which the American crow (*Corvus brachyrhynchos*, 81), blue jay (*Cyanocitta cristata*, 26), and black-billed magpie (*Pica hudsonia*, 1) were identified as hosts in these data. The remaining avian hosts include *Falco sparverius* (1), *Poecile atricapillus* (1), *Quiscalus quiscula* (1), *Accipiter cooperii* (1), *Buteo jamaicensis* (1), *Zenaida macroura* (1), *Mimus polyglottos* (2), and Loriidae (1). We provide the full list of sequences and their metadata as Supplementary Material.

#### Bayesian Phylogeographic GLM

The phylogeographic model assumes that the location of each ancestral lineage is governed by a continuous-time Markov chain (CTMC) process that runs along the branches of an unknown phylogeny that is informed through sequence data. The infinitesimal rate matrix of the among-location CTMC process is parameterized as a log-linear GLM of predictors of interest^5^ to determine the probability of inclusion and the contribution of these predictor variables. Here, we selected predictors of interest to parameterize this rate matrix and estimate posterior probabilities of all 2^*P*^ linear models via a Bayesian stochastic search variable selection (BSSVS) procedure^5^, where *P* is the number of predictors. We specified a 50% prior probability that no predictor is included in the model and evaluated the support of each predictor via Bayes factors (BFs), where we consider any predictor with BF >3.0 to be supported for inclusion in the model^27^ following similar studies^5,7–9^.

#### Levels of predictor aggregation

**Figure 5:**
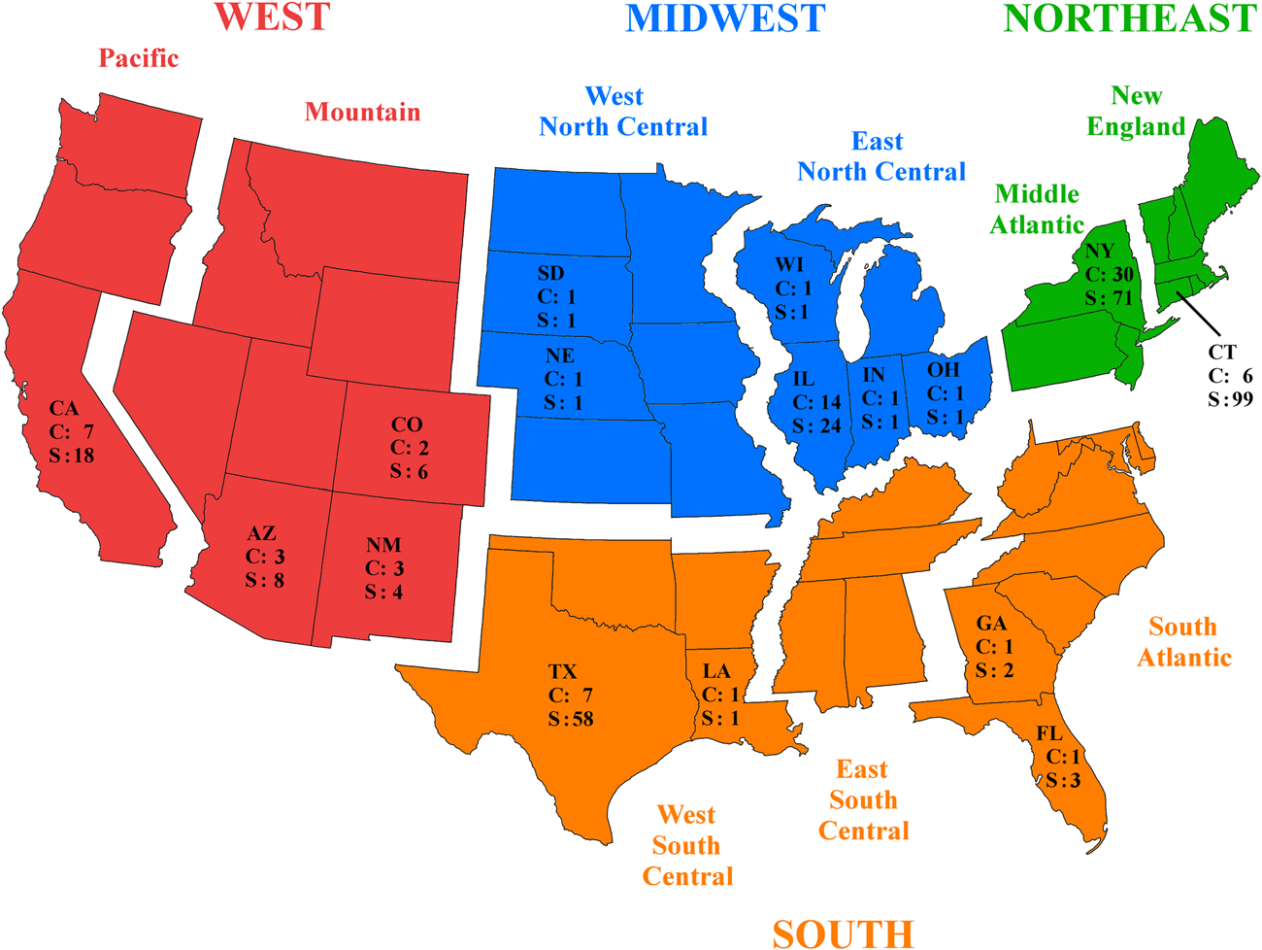
Visualization of the discrete state partitions. The four colors represent the discrete locations for the CBR model, which are further discretized into nine CBS locations, 16 states, and 80 counties. No samples were available for the East South Central subdivision at the CBS level. Each state is annotated with its number of unique sampled counties (C) and number of sequences (S). The Midwest, Northeast, South, and West regional models are county-level aggregations encapsulated by the *K* counties in their respective CBR. We utilized a blank map of the U.S. from ArcMap v10.3 and created the figure using GIMP v2.8^38^.

As we wish to investigate the differences in support for the GLM predictors when the sampling locations are specified with more or less resolution, we used four levels of aggregation for each predictor: USCB regions (CBR, *K* = 4), USCB subdivisions (CBS, *K* = 8), state (*K* = 16), and county (*K* = 80). At each level, we assume that the sampling location of each virus is known to one of the *K* discrete states. We define the aggregation levels as ranging from “most uncertain” (CBR) to “least uncertain” (county) as knowledge of the sampling location increases. We obtained internal latitude and longitude coordinates for each state in the contiguous U.S., including the District of Columbia, as well as every known sampled county from the USCB. For the CBR and CBS aggregations, the geospatial reference is the mean latitude and longitude of the states in the respective boundaries. In order to investigate whether regional dynamics of WNV match those at the national level for the four aforementioned aggregations, we also include a county-level aggregation for the sequences collected in each of the four CBR discrete states. That is, we selected the sequences from the four USCB regions, Midwest (n = 29), Northeast (n = 170), South (n = 64), and West (n = 36), and completed a regional analysis for each at the county-level aggregation. In Fig. 5, we provide a map of the discrete states in each level of aggregation and we detail the metadata for each sequence in our study as Supplementary Material.

### GLM Predictors

We identified predictor data for each discrete state to represent the WNV epidemic in the U.S. from 1999–2012. Although the exact dates of the estimates for each predictor vary, each was accurate as of one specific point in time during the years of our study.

#### Distance (DS)

We obtained a centroid latitude and longitude of each state and county from the USCB MAF/TIGER database. We calculated the pairwise distance from each location to the next using these coordinates for the state and county aggregations, respectively. For the CBR and CBS aggregations, we calculated the mean internal latitude and longitude for each of the states in the defined area. We used these means as the centroid coordinates for each area and calculated pairwise distances between them.

#### Population density (PD)

We obtained human census data from the USCB for the most recent full census in 2010. We obtained population data at both the state and county levels. For the CBR and CBS, we summed the total population in the respective states and divided by their total land area to obtain a density. For the state and county aggregations, we divided the population per sampled location by its land area to obtain a density.

#### Case counts (CC)

We obtained data on the number of cases per state from the Centers for Disease Control and Prevention’s (CDC) ArboNET surveillance program^13^. These data reflect the cumulative number of human cases per year from 1999–2012 at the state level. The predictor for the CBR and CBS aggregations reflect the total number of human cases in the defined states during this period. For the county aggregation, our point estimate assumes that the county observed a number of cases proportional to its population within the state. These data round to zero for Concho County (Texas) and Wilkinson County (Georgia), so they were changed to a value of one to ensure positivity for the log transformation.

#### Unvaccinated Horses (UH)

The American Horse Council Foundation completed the most comprehensive horse census in the U.S. during the time period of our study. This survey counted the number of horses per state, and includes horses found on farms, private homes, and those in the racing, showing, and recreation industries as of 2005^17^. As data was only available for the state level, we assumed that these animals were uniformly distributed across the entire state. Thus, the state aggregation encapsulates all horses estimated to be in the state, and the county level reflects the expected number of horses given the county’s land area. We used the sum of horses in each state for the CBR and CBS aggregations. Furthermore, the Animal and Plant Health Inspection Service (APHIS) of the U.S. Department of Agriculture (USDA) provided estimates of equid vaccination practices during the 2005 calendar year^18^. This study surveyed equid owners in 28 states, 12 of which were included in this study. The survey provided average vaccination rates of resident equids, discretized into four regions: South, Northeast, West, and Central. For the CBR and CBS aggregations, we matched each region to its most appropriate of the four USDA regions. At the state level, we used the average rates per region in the USDA study for the 12 states in this study and assumed the remaining four states to be part of the most proximal geographic region. At the county level, we use the same vaccination rate as its corresponding state. Given the horse census and the vaccination rates, we use the expected number of unvaccinated horses in each discrete state as the predictor at each level of aggregation.

#### Corvidae Counts (CA)

Of the 299 sequences used in this study, we identified 118 that were isolated from avian hosts, including 11 unique species, however 104 of these sequences (92%) were from birds of the *Corvidae* family. The Cornell Lab of Ornithology^28^ provides a collection of census data obtained by birders throughout the world and can be selected by species, geographic region, and time. We obtained the total number of observations of the three *Corvidae* hosts (*Corvus brachyrhynchos*, *Cyanocitta cristata*, and *Pica hudsonia*) during 1999–2012 for each respective discrete state at the CBR, CBS, state, and county levels, as well as the total number of reports. To account for potential biases in the reporting of birders in various locations, we divided the cumulative counts of the three species by the cumulative number of reports to obtain an expected number of *Corvidae* sightings per observation in each discrete state and used this as the predictor at each level.

#### Wetlands Area (WL)

We obtained GIS shapefiles of each of the states in this analysis from the U.S. Fish and Wildlife Service^29^. These files contain the wetlands polygon data for each state, and we extracted the total area of wetlands for the state level using ArcMap v10 (ESRI, Inc., Redlands, CA, USA). For the CBR and CBS aggregations, we used the sum of each state’s wetlands to obtain the total wetlands per defined area. For the county level, we obtained the map of counties in each state from the USCB MAF/TIGER database and extracted all instances of wetlands contained in the respective counties. We divided each wetlands area by the total land area of each discrete state to obtain percentage wetlands cover and used this as the predictor point estimate at each level of aggregation.

#### Temperature (TP) and precipitation (PC)

We obtained temperature and precipitation data from the 30-year normal datasets (1981–2010) provided by the National Climatic Data Center of the National Oceanic and Atmospheric Administration (NOAA)^30^. At the CBR, CBS, state, and county levels, we extracted the average annual temperature and precipitation data from each NOAA station in the respective areas. The temperature and precipitation predictors thus reflect the average 30-year normal observed by all stations in each discrete state. At the county level, there were several instances where either no NOAA station existed within the county boundaries or the station(s) in the county did not contain normal temperature or precipitation data. In these instances, we used the most proximal station within that state to the county’s centroid coordinates that contained both temperature and precipitation normal.

We log-transformed and standardized all predictor data and created a separate predictor from both discrete state of origin and discrete state of destination, with the exception of the distance predictor, for a total of 15 predictors. We summarize the distributions of the predictors at level of aggregation in Fig. 5.

### BEAST Analysis

We specified a generalized time reversible substitution model following previous WNV studies^11,12,16,22,31,32^, also including invariant sites and a gamma heterogeneity (GTR + I + G) on our sequences. We set an uncorrelated lognormal relaxed molecular clock^33^ following previous studies^11,22,34^ with an initial mutation rate of 0.001 substitutions per site per year and specified a Bayesian Skyline prior^35^. For each discrete space partitioning, we specified a phylogeographic GLM^5^ using the respective predictor data at each level of aggregation. We evaluated each using the BEAST v1.8.4 software package^36^ with a chain length of 250 M and sampling every 25,000 steps for the CBR, CBS, state, and national county-level models. For the four regional models, we specified a chain length of 150 M with sampling every 15,000 steps. We used TreeAnnotator v1.8.4 to construct a maximum clade credibility (MCC) tree for each model after discarding the first 10% of trees as burn-in and annotated the trees using FigTree v1.4.2. We obtained the mean posterior probability of inclusion, BF support, and the contribution of each GLM predictor for each model using Tracer v1.6.

#### Predictor variance correlations

From each model and for each predictor, we extracted the standard deviation of the inclusion probability, the standard deviation of the regression coefficient, and the upper and lower bounds of the regression coefficient’s HPD. We used the “geom_smooth” function in the “ggplot” package in R v3.3.1^37^ to visualize correlations between the variance of predictor point estimates and variance in posterior support. We used the “lm” function to obtain these R^2^ values for each individual model and with all models pooled together.

### Data availability

We have made all sequence metadata, FASTA alignments, XML files, MCC phylogenies, and Bayesian Skyline plots freely available at https://figshare.com/projects/WNV_GLM_Aggregation_Study/19201.

## Electronic supplementary material

Supplementary Material

## References

[1] Slatkin M, Maddison WP (1989). A cladistic measure of gene flow inferred from the phylogenies of alleles. Genetics.

[2] Lemey P, Rambaut A, Drummond AJ, Suchard MA (2009). Bayesian phylogeography finds its roots. PLoS Comput Biol.

[3] Kuo L, Mallick B (1998). Variable Selection for Regression Models. The Indian Journal of Statistics, Series B.

[4] Chipman H, George EI, McCulloch RE (2001). The Practical Implementation of Bayesian Model Selection. IMS Lecture Notes - Monograph Series.

[5] Lemey P (2014). Unifying viral genetics and human transportation data to predict the global transmission dynamics of human influenza H3N2. PLoS Pathog.

[6] Nunes MR (2014). Air travel is associated with intracontinental spread of dengue virus serotypes 1–3 in Brazil. PLoS Negl Trop Dis.

[7] Magee D, Suchard MA, Scotch M (2017). Bayesian phylogeography of influenza A/H3N2 for the 2014–15 season in the United States using three frameworks of ancestral state reconstruction. PLoS Comput Biol.

[8] Magee D, Beard R, Suchard MA, Lemey P, Scotch M (2015). Combining phylogeography and spatial epidemiology to uncover predictors of H5N1 influenza A virus diffusion. Arch Virol.

[9] Graf T (2015). Contribution of Epidemiological Predictors in Unraveling the Phylogeographic History of HIV-1 Subtype C in Brazil. J Virol.

[10] Rambaut A (2008). The genomic and epidemiological dynamics of human influenza A virus. Nature.

[11] Pybus OG (2012). Unifying the spatial epidemiology and molecular evolution of emerging epidemics. Proc Natl Acad Sci USA.

[12] Mann BR, McMullen AR, Swetnam DM, Barrett AD (2013). Molecular epidemiology and evolution of West Nile virus in North America. Int J Environ Res Public Health.

[13] CDC. *West Nile virus disease cases reported to CDC by state of residence, 1999*–2014, https://www.cdc.gov/westnile/resources/pdfs/data/2-west-nile-virus-disease-cases-reported-to-cdc-by-state_1999-2014_06042015.pdf (2015).

[14] Sardelis MR, Turell MJ, Dohm DJ, O’Guinn ML (2001). Vector competence of selected North American Culex and Coquillettidia mosquitoes for West Nile virus. Emerg Infect Dis.

[15] WHO. *West Nile virus*, http://www.who.int/mediacentre/factsheets/fs354/en/ (2011).

[16] Di Giallonardo F (2015). Fluid Spatial Dynamics of West Nile Virus in the United States: Rapid Spread in a Permissive Host Environment. J Virol.

[17] American Horse Council, https://www.usef.org/media/press-releases/645_most-comprehensive-horse-study-ever-reveals-a-nearly–billion-impact-on-the-us-economy (2005).

[18] APHIS-USDA. *Vaccination Practices on U.S. Equine Operations*, https://www.aphis.usda.gov/animal_health/nahms/equine/downloads/equine05/Equine05_is_Vaccination.pdf (2006).

[19] Komar N (2000). West Nile viral encephalitis. Rev Sci Tech.

[20] Williams, C. A. *West Nile Virus in Horses: Frequently Asked Questions*, https://esc.rutgers.edu/fact_sheet/west-nile-virus-in-horses-frequently-asked-questions/ (2004).

[21] AAEP. *West Nile Virus*, https://aaep.org/horsehealth/west-nile-virus (2017).

[22] Anez G (2013). Evolutionary dynamics of West Nile virus in the United States, 1999–2011: phylogeny, selection pressure and evolutionary time-scale analysis. PLoS Negl Trop Dis.

[23] Tahsin T (2016). A high-precision rule-based extraction system for expanding geospatial metadata in GenBank records. J Am Med Inform Assoc.

[24] Scotch M (2011). Enhancing phylogeography by improving geographical information from GenBank. J Biomed Inform.

[25] Pickett BE (2012). ViPR: an open bioinformatics database and analysis resource for virology research. Nucleic Acids Res.

[26] Armstrong PM (2011). Molecular evolution of West Nile virus in a northern temperate region: Connecticut, USA 1999-2008. Virology.

[27] Kass RE, Raftery A (1995). Bayes factors. Journal of the American Statistical Association.

[28] Sullivan BL (2009). eBird: A citizen-based bird observation network in the biological sciences. Biological Conservation.

[29] USFWS. *Geospatial Services*, https://www.fws.gov/gis (2014).

[30] Arguez A (2012). NOAA’s 1981–2010 U.S. Climate Normals: An Overview. Bulletin of the American Meteorological Society.

[31] Lopez RH, Soto SU, Gallego-Gomez JC (2015). Evolutionary relationships of West Nile virus detected in mosquitoes from a migratory bird zone of Colombian Caribbean. Virol J.

[32] Duggal NK (2014). Evidence for co-evolution of West Nile Virus and house sparrows in North America. PLoS Negl Trop Dis.

[33] Drummond AJ, Ho SY, Phillips MJ, Rambaut A (2006). Relaxed phylogenetics and dating with confidence. PLoS Biol.

[34] Ciccozzi M (2013). Epidemiological history and phylogeography of West Nile virus lineage 2. Infect Genet Evol.

[35] Drummond AJ, Rambaut A, Shapiro B, Pybus OG (2005). Bayesian coalescent inference of past population dynamics from molecular sequences. Mol Biol Evol.

[36] Drummond AJ, Suchard MA, Xie D, Rambaut A (2012). Bayesian phylogenetics with BEAUti and the BEAST 1.7. Mol Biol Evol.

[37] R Core Team. *R: A language and environment for statistical computing*, http://www.R-project.org/ (2014).

[38] GIMP. *Downloads*, https://www.gimp.org/downloads/ (2017).

