# Supplementary material for: The Effects of Sampling Location and Predictor Point Estimate Certainty on Posterior Support in Bayesian Phylogeographic Generalized Linear Models
Source: Sci Rep. 2018 Apr 12;8:5905. doi: 10.1038/s41598-018-24264-8 (PMC5897398; doi:10.1038/s41598-018-24264-8)
Supplement: Supplementary file 1 — Supplementary Material [file 41598_2018_24264_MOESM1_ESM.docx]

**SUPPLEMENTARY MATERIAL**

**Manuscript Title**

The effects of sampling location and predictor point estimate certainty on posterior support in Bayesian phylogeographic generalized linear models

**Authors and Affiliations**

Daniel Magee^1,2^, Jesse E. Taylor^3,4^, Matthew Scotch^1,2^

^1^ Department of Biomedical Informatics, Arizona State University, Tempe, Arizona, United

States of America

^2^ Biodesign Center for Environmental Security, Arizona State University, Tempe, Arizona,

United States of America

^3^ School of Mathematical and Statistical Sciences, Arizona State University, Tempe, Arizona,

United States of America

^4^ School of Life Sciences, Arizona State University, Tempe, Arizona, United States of America

* Corresponding author

E-mail: [matthew.scotch@asu.edu](mailto:matthew.scotch@asu.edu)

**Table S1.** Metadata for the 299 sequences used in this study.

| **Accession** | **Host** | **CBR** | **CBS** | **State** | **County** | **Year** |
| --- | --- | --- | --- | --- | --- | --- |
| DQ164186 | *Corvus brachyrhynchos* | Northeast | Middle Atlantic | New York | Queens | 2002 |
| DQ164187 | *Corvus brachyrhynchos* | Northeast | Middle Atlantic | New York | Broome | 2002 |
| DQ164188 | *Corvus brachyrhynchos* | Northeast | Middle Atlantic | New York | Westchester | 2003 |
| DQ164189 | *Corvus brachyrhynchos* | Northeast | Middle Atlantic | New York | Albany | 2003 |
| DQ164190 | *Corvus brachyrhynchos* | Northeast | Middle Atlantic | New York | Suffolk | 2003 |
| DQ164191 | *Corvus brachyrhynchos* | Northeast | Middle Atlantic | New York | Chautauqua | 2003 |
| DQ164192 | *Corvus brachyrhynchos* | Northeast | Middle Atlantic | New York | Rockland | 2003 |
| DQ164193 | *Corvus brachyrhynchos* | Northeast | Middle Atlantic | New York | Clinton | 2002 |
| DQ164194 | *Corvus brachyrhynchos* | Northeast | Middle Atlantic | New York | Suffolk | 2001 |
| DQ164195 | *Culex pipiens/restuans* | Northeast | Middle Atlantic | New York | Nassau | 2002 |
| DQ164196 | *Homo sapiens* | South | South Atlantic | Georgia | Wilkinson | 2002 |
| DQ164197 | *Homo sapiens* | South | South Atlantic | Georgia | Wilkinson | 2002 |
| DQ164198 | *Homo sapiens* | South | West South Central | Texas | Concho | 2002 |
| DQ164199 | *Homo sapiens* | South | West South Central | Texas | Concho | 2003 |
| DQ164200 | *Homo sapiens* | Midwest | East North Central | Indiana | Hendricks | 2002 |
| DQ164201 | *Homo sapiens* | West | Mountain | Arizona | Yavapai | 2004 |
| DQ164202 | *Homo sapiens* | Midwest | East North Central | Ohio | Licking | 2002 |
| DQ164203 | *Pica hudsonia* | West | Mountain | Colorado | Park | 2003 |
| DQ164204 | *Buteo jamaicensis* | West | Mountain | Colorado | Park | 2003 |
| DQ164205 | *Homo sapiens* | South | West South Central | Texas | Concho | 2002 |
| DQ164206 | *Cyanocitta cristata* | South | West South Central | Texas | Harris | 2004 |
| DQ431693 | *Homo sapiens* | South | West South Central | Texas | Randall | 2003 |
| DQ431695 | *Homo sapiens* | Midwest | East North Central | Illinois | Cook | 2003 |
| DQ431696 | *Homo sapiens* | Midwest | East North Central | Wisconsin | Milwaukee | 2003 |
| DQ431697 | *Homo sapiens* | South | South Atlantic | Florida | Hillsborough | 2003 |
| DQ431698 | *Homo sapiens* | South | South Atlantic | Florida | Hillsborough | 2003 |
| DQ431699 | *Homo sapiens* | South | South Atlantic | Florida | Hillsborough | 2003 |
| DQ431700 | *Homo sapiens* | West | Pacific | California | San Francisco | 2004 |
| DQ431701 | *Homo sapiens* | West | Mountain | Colorado | Mesa | 2004 |
| DQ431702 | *Homo sapiens* | West | Mountain | Colorado | Mesa | 2004 |
| DQ431703 | *Homo sapiens* | West | Mountain | Colorado | Mesa | 2004 |
| DQ431704 | *Homo sapiens* | West | Mountain | Colorado | Mesa | 2004 |
| DQ431705 | *Homo sapiens* | Midwest | West North Central | South Dakota | Pennington | 2004 |
| DQ431706 | *Homo sapiens* | West | Mountain | New Mexico | Sandoval | 2004 |
| DQ431707 | *Homo sapiens* | West | Mountain | New Mexico | Sandoval | 2004 |
| DQ431708 | *Homo sapiens* | West | Pacific | California | San Diego | 2004 |
| DQ431709 | *Homo sapiens* | West | Pacific | California | San Bernardino | 2004 |
| DQ431710 | *Homo sapiens* | West | Pacific | California | Orange | 2004 |
| DQ431711 | *Homo sapiens* | West | Mountain | Arizona | Maricopa | 2004 |
| DQ431712 | *Homo sapiens* | West | Mountain | Arizona | Maricopa | 2004 |
| EF530047 | *Corvus brachyrhynchos* | Northeast | Middle Atlantic | New York | Richmond | 2000 |
| EF657887 | *Corvus brachyrhynchos* | Northeast | Middle Atlantic | New York | Richmond | 2000 |
| FJ151394 | *Corvus brachyrhynchos* | Northeast | Middle Atlantic | New York | New York | 1999 |
| FJ527738 | *Cyanocitta cristata* | South | West South Central | Louisiana | Jefferson | 2001 |
| GQ507468 | *Homo sapiens* | South | West South Central | Texas | El Paso | 2005 |
| GQ507469 | *Homo sapiens* | West | Mountain | New Mexico | Dona Ana | 2005 |
| GQ507470 | *Homo sapiens* | South | West South Central | Texas | El Paso | 2006 |
| GQ507471 | *Homo sapiens* | South | West South Central | Texas | El Paso | 2007 |
| GQ507472 | *Homo sapiens* | West | Pacific | California | Orange | 2003 |
| GQ507473 | *Homo sapiens* | West | Pacific | California | Los Angeles | 2004 |
| GQ507474 | *Homo sapiens* | West | Pacific | California | San Bernardino | 2004 |
| GQ507475 | *Homo sapiens* | West | Pacific | California | San Bernardino | 2005 |
| GQ507476 | *Homo sapiens* | West | Pacific | California | San Bernardino | 2005 |
| GQ507477 | *Homo sapiens* | West | Pacific | California | Los Angeles | 2005 |
| GQ507478 | *Homo sapiens* | West | Pacific | California | Los Angeles | 2005 |
| GQ507479 | *Homo sapiens* | West | Mountain | Arizona | Pima | 2005 |
| GQ507480 | *Homo sapiens* | West | Pacific | California | Los Angeles | 2005 |
| GQ507481 | *Homo sapiens* | Midwest | West North Central | Nebraska | Douglas | 2006 |
| GQ507482 | *Homo sapiens* | West | Mountain | Arizona | Pima | 2006 |
| GQ507483 | *Homo sapiens* | West | Pacific | California | Los Angeles | 2007 |
| GQ507484 | *Homo sapiens* | West | Pacific | California | Los Angeles | 2007 |
| GU827998 | *Cyanocitta cristata* | South | West South Central | Texas | Harris | 2002 |
| GU827999 | *Cyanocitta cristata* | South | West South Central | Texas | Montgomery | 2003 |
| GU828000 | *Cyanocitta cristata* | South | West South Central | Texas | Harris | 2003 |
| GU828001 | *Culex quinquefasciatus* | South | West South Central | Texas | Harris | 2003 |
| GU828002 | *Culex quinquefasciatus* | South | West South Central | Texas | Harris | 2003 |
| GU828003 | *Zenaida macroura* | South | West South Central | Texas | Jefferson | 2003 |
| GU828004 | *Cyanocitta cristata* | South | West South Central | Texas | Montgomery | 2003 |
| HM488114 | *Aedes cinereus* | Northeast | New England | Connecticut | Fairfield | 2002 |
| HM488115 | *Culex salinarius* | Northeast | New England | Connecticut | Fairfield | 2005 |
| HM488116 | *Culex pipiens* | Northeast | New England | Connecticut | Fairfield | 2005 |
| HM488117 | *Ochlerotatus triseriatus* | Northeast | New England | Connecticut | Fairfield | 2005 |
| HM488118 | *Culex pipiens* | Northeast | New England | Connecticut | Fairfield | 2005 |
| HM488119 | *Culex pipiens* | Northeast | New England | Connecticut | Fairfield | 2005 |
| HM488120 | *Culex pipiens* | Northeast | New England | Connecticut | Fairfield | 2005 |
| HM488121 | *Culex pipiens* | Northeast | New England | Connecticut | Fairfield | 2005 |
| HM488125 | *Corvus brachyrhynchos* | Northeast | New England | Connecticut | Fairfield | 1999 |
| HM488126 | *Corvus brachyrhynchos* | Northeast | New England | Connecticut | Fairfield | 1999 |
| HM488127 | *Corvus brachyrhynchos* | Northeast | New England | Connecticut | Fairfield | 1999 |
| HM488128 | *Corvus brachyrhynchos* | Northeast | New England | Connecticut | Fairfield | 1999 |
| HM488129 | *Culex salinarius* | Northeast | New England | Connecticut | New Haven | 2000 |
| HM488130 | *Culex salinarius* | Northeast | New England | Connecticut | New Haven | 2000 |
| HM488131 | *Culex pipiens* | Northeast | New England | Connecticut | New Haven | 2000 |
| HM488132 | *Culiseta melanura* | Northeast | New England | Connecticut | Fairfield | 2000 |
| HM488133 | *Culex pipiens* | Northeast | New England | Connecticut | Fairfield | 2001 |
| HM488134 | *Ochlerotatus sollicitans* | Northeast | New England | Connecticut | Fairfield | 2001 |
| HM488135 | *Ochlerotatus cantator* | Northeast | New England | Connecticut | Fairfield | 2001 |
| HM488136 | *Culex restuans* | Northeast | New England | Connecticut | Fairfield | 2001 |
| HM488137 | *Culex pipiens* | Northeast | New England | Connecticut | Fairfield | 2002 |
| HM488138 | *Culex restuans* | Northeast | New England | Connecticut | Fairfield | 2003 |
| HM488139 | *Culex salinarius* | Northeast | New England | Connecticut | Fairfield | 2003 |
| HM488140 | *Aedes vexans* | Northeast | New England | Connecticut | Fairfield | 2003 |
| HM488141 | *Culex pipiens* | Northeast | New England | Connecticut | Fairfield | 2003 |
| HM488142 | *Ochlerotatus triseriatus* | Northeast | New England | Connecticut | Fairfield | 2004 |
| HM488143 | *Aedes cinereus* | Northeast | New England | Connecticut | Fairfield | 2004 |
| HM488144 | *Culex restuans* | Northeast | New England | Connecticut | Fairfield | 2004 |
| HM488145 | *Aedes vexans* | Northeast | New England | Connecticut | Fairfield | 2004 |
| HM488146 | *Psorophora ferox* | Northeast | New England | Connecticut | Fairfield | 2004 |
| HM488147 | *Culex salinarius* | Northeast | New England | Connecticut | Fairfield | 2004 |
| HM488148 | *Culex pipiens* | Northeast | New England | Connecticut | Fairfield | 2004 |
| HM488149 | *Ochlerotatus cantator* | Northeast | New England | Connecticut | Fairfield | 2005 |
| HM488150 | *Culex pipiens* | Northeast | New England | Connecticut | Fairfield | 2005 |
| HM488151 | *Culex pipiens* | Northeast | New England | Connecticut | Fairfield | 2005 |
| HM488152 | *Aedes vexans* | Northeast | New England | Connecticut | Fairfield | 2005 |
| HM488153 | *Culex pipiens* | Northeast | New England | Connecticut | Fairfield | 2005 |
| HM488154 | *Culex pipiens* | Northeast | New England | Connecticut | Fairfield | 2005 |
| HM488155 | *Culex restuans* | Northeast | New England | Connecticut | Fairfield | 2006 |
| HM488156 | *Culex pipiens* | Northeast | New England | Connecticut | Fairfield | 2006 |
| HM488157 | *Culex pipiens* | Northeast | New England | Connecticut | Fairfield | 2006 |
| HM488158 | *Culex pipiens* | Northeast | New England | Connecticut | Fairfield | 2006 |
| HM488159 | *Culex salinarius* | Northeast | New England | Connecticut | Fairfield | 2006 |
| HM488160 | *Culex pipiens* | Northeast | New England | Connecticut | Fairfield | 2006 |
| HM488161 | *Culex pipiens* | Northeast | New England | Connecticut | Fairfield | 2007 |
| HM488162 | *Culex pipiens* | Northeast | New England | Connecticut | Fairfield | 2007 |
| HM488163 | *Culex pipiens* | Northeast | New England | Connecticut | Fairfield | 2007 |
| HM488164 | *Culex pipiens* | Northeast | New England | Connecticut | Fairfield | 2007 |
| HM488165 | *Culex pipiens* | Northeast | New England | Connecticut | Fairfield | 2007 |
| HM488166 | *Culex pipiens* | Northeast | New England | Connecticut | Fairfield | 2008 |
| HM488167 | *Culex pipiens* | Northeast | New England | Connecticut | Fairfield | 2008 |
| HM488168 | *Culex pipiens* | Northeast | New England | Connecticut | Fairfield | 2008 |
| HM488169 | *Culex pipiens* | Northeast | New England | Connecticut | Fairfield | 2008 |
| HM488170 | *Culex pipiens* | Northeast | New England | Connecticut | Fairfield | 2008 |
| HM488171 | *Culex restuans* | Northeast | New England | Connecticut | Fairfield | 2003 |
| HM488172 | *Ochlerotatus sticticus* | Northeast | New England | Connecticut | Fairfield | 2003 |
| HM488173 | *Culex restuans* | Northeast | New England | Connecticut | New Haven | 2003 |
| HM488174 | *Culex salinarius* | Northeast | New England | Connecticut | New Haven | 2003 |
| HM488175 | *Culex restuans* | Northeast | New England | Connecticut | Hartford | 2003 |
| HM488176 | *Culex salinarius* | Northeast | New England | Connecticut | New Haven | 2003 |
| HM488177 | *Corvus brachyrhynchos* | Midwest | East North Central | Illinois | Cook | 2002 |
| HM488178 | *Corvus brachyrhynchos* | Midwest | East North Central | Illinois | Cook | 2002 |
| HM488180 | *Corvus brachyrhynchos* | Midwest | East North Central | Illinois | Cook | 2002 |
| HM488181 | *Corvus brachyrhynchos* | Midwest | East North Central | Illinois | Iroquois | 2002 |
| HM488182 | *Corvus brachyrhynchos* | Midwest | East North Central | Illinois | Clinton | 2002 |
| HM488183 | *Cyanocitta cristata* | Midwest | East North Central | Illinois | Douglas | 2002 |
| HM488184 | *Cyanocitta cristata* | Midwest | East North Central | Illinois | Moultrie | 2002 |
| HM488185 | *Cyanocitta cristata* | Midwest | East North Central | Illinois | Cook | 2003 |
| HM488186 | *Corvus brachyrhynchos* | Midwest | East North Central | Illinois | Champaign | 2003 |
| HM488188 | *Corvus brachyrhynchos* | Midwest | East North Central | Illinois | Vermilion | 2004 |
| HM488189 | *Cyanocitta cristata* | Midwest | East North Central | Illinois | Will | 2004 |
| HM488190 | *Corvus brachyrhynchos* | Midwest | East North Central | Illinois | Cook | 2004 |
| HM488191 | *Corvus brachyrhynchos* | Midwest | East North Central | Illinois | Cook | 2004 |
| HM488192 | *Corvus brachyrhynchos* | Midwest | East North Central | Illinois | Rock Island | 2005 |
| HM488193 | *Corvus brachyrhynchos* | Midwest | East North Central | Illinois | St. Clair | 2005 |
| HM488194 | *Corvus brachyrhynchos* | Midwest | East North Central | Illinois | Lake | 2005 |
| HM488195 | *Cyanocitta cristata* | Midwest | East North Central | Illinois | Kendall | 2005 |
| HM488196 | *Corvus brachyrhynchos* | Midwest | East North Central | Illinois | Cook | 2005 |
| HM488197 | *Corvus brachyrhynchos* | Midwest | East North Central | Illinois | McHenry | 2005 |
| HM488203 | *Corvus brachyrhynchos* | Northeast | Middle Atlantic | New York | Putnam | 2008 |
| HM488204 | *Corvus brachyrhynchos* | Northeast | Middle Atlantic | New York | Suffolk | 2008 |
| HM488205 | *Corvus brachyrhynchos* | Northeast | Middle Atlantic | New York | Albany | 2008 |
| HM488206 | *Corvus brachyrhynchos* | Northeast | Middle Atlantic | New York | Erie | 2008 |
| HM488207 | *Cyanocitta cristata* | Northeast | Middle Atlantic | New York | Nassau | 2008 |
| HM488208 | *Culex salinarius* | Northeast | New England | Connecticut | Fairfield | 2002 |
| HM488209 | *Ochlerotatus sticticus* | Northeast | New England | Connecticut | Fairfield | 2003 |
| HM488210 | *Culiseta melanura* | Northeast | New England | Connecticut | New Haven | 2003 |
| HM488212 | *Culex salinarius* | Northeast | New England | Connecticut | New Haven | 2003 |
| HM488213 | *Culex restuans* | Northeast | New England | Connecticut | Fairfield | 2003 |
| HM488214 | *Culex pipiens* | Northeast | New England | Connecticut | Fairfield | 2003 |
| HM488215 | *Culiseta melanura* | Northeast | New England | Connecticut | Fairfield | 2003 |
| HM488216 | *Culiseta melanura* | Northeast | New England | Connecticut | New London | 2003 |
| HM488217 | *Culex salinarius* | Northeast | New England | Connecticut | New Haven | 2003 |
| HM488218 | *Culex pipiens* | Northeast | New England | Connecticut | Fairfield | 2003 |
| HM488219 | *Culex pipiens* | Northeast | New England | Connecticut | Hartford | 2003 |
| HM488220 | *Culex salinarius* | Northeast | New England | Connecticut | New Haven | 2003 |
| HM488221 | *Culiseta melanura* | Northeast | New England | Connecticut | New London | 2003 |
| HM488222 | *Culiseta melanura* | Northeast | New England | Connecticut | New London | 2003 |
| HM488223 | *Culiseta melanura* | Northeast | New England | Connecticut | Fairfield | 2003 |
| HM488224 | *Culiseta melanura* | Northeast | New England | Connecticut | Fairfield | 2003 |
| HM488225 | *Aedes cinereus* | Northeast | New England | Connecticut | New Haven | 2003 |
| HM488226 | *Culex pipiens* | Northeast | New England | Connecticut | New Haven | 2003 |
| HM488227 | *Culex restuans* | Northeast | New England | Connecticut | New Haven | 2003 |
| HM488228 | *Culex salinarius* | Northeast | New England | Connecticut | New Haven | 2003 |
| HM488229 | *Psorophora ferox* | Northeast | New England | Connecticut | New Haven | 2003 |
| HM488230 | *Culex salinarius* | Northeast | New England | Connecticut | Windham | 2003 |
| HM488231 | *Culiseta melanura* | Northeast | New England | Connecticut | Middlesex | 2003 |
| HM488232 | *Culiseta melanura* | Northeast | New England | Connecticut | Middlesex | 2003 |
| HM488233 | *Aedes vexans* | Northeast | New England | Connecticut | New Haven | 2003 |
| HM488234 | *Culex salinarius* | Northeast | New England | Connecticut | New Haven | 2003 |
| HM488235 | *Culiseta melanura* | Northeast | New England | Connecticut | Fairfield | 2003 |
| HM488236 | *Culiseta melanura* | Northeast | New England | Connecticut | Middlesex | 2003 |
| HM488237 | *Corvus brachyrhynchos* | Northeast | Middle Atlantic | New York | Onondaga | 2008 |
| HM488238 | *Corvus brachyrhynchos* | Northeast | Middle Atlantic | New York | Onondaga | 2008 |
| HM488239 | *Corvus brachyrhynchos* | Northeast | Middle Atlantic | New York | Putnam | 2008 |
| HM488240 | *Cyanocitta cristata* | Northeast | Middle Atlantic | New York | Suffolk | 2008 |
| HM488241 | *Corvus brachyrhynchos* | Northeast | Middle Atlantic | New York | Niagara | 2008 |
| HM488242 | *Poecile atricapilla* | Northeast | Middle Atlantic | New York | Dutchess | 2008 |
| HM488243 | *Corvus brachyrhynchos* | Northeast | Middle Atlantic | New York | Suffolk | 2008 |
| HM488244 | *Falco sparverius* | Northeast | Middle Atlantic | New York | Erie | 2008 |
| HM488245 | *Corvus brachyrhynchos* | Northeast | Middle Atlantic | New York | Putnam | 2008 |
| HM488246 | *Corvus brachyrhynchos* | Northeast | Middle Atlantic | New York | Kings | 2001 |
| HM488247 | *Corvus brachyrhynchos* | Northeast | Middle Atlantic | New York | New York | 2001 |
| HM488248 | *Corvus brachyrhynchos* | Northeast | Middle Atlantic | New York | Herkimer | 2001 |
| HM488249 | *Corvus brachyrhynchos* | Northeast | Middle Atlantic | New York | Onondaga | 2001 |
| HM488250 | *Corvus brachyrhynchos* | Northeast | Middle Atlantic | New York | Broome | 2003 |
| HM488251 | *Corvus brachyrhynchos* | Northeast | Middle Atlantic | New York | Cortland | 2003 |
| HM488252 | *Corvus brachyrhynchos* | Northeast | Middle Atlantic | New York | Onondaga | 2005 |
| HM756648 | *Ochlerotatus trivittatus* | Northeast | New England | Connecticut | Fairfield | 2002 |
| HM756649 | *Culex pipiens* | Northeast | New England | Connecticut | Fairfield | 2006 |
| HM756650 | *Culex salinarius* | Northeast | New England | Connecticut | New Haven | 2003 |
| HM756651 | *Ochlerotatus trivittatus* | Northeast | New England | Connecticut | Fairfield | 2003 |
| HM756652 | *Aedes cinereus* | Northeast | New England | Connecticut | Middlesex | 2003 |
| HM756653 | *Culex pipiens* | Northeast | New England | Connecticut | Middlesex | 2003 |
| HM756654 | *Culex salinarius* | Northeast | New England | Connecticut | Fairfield | 2003 |
| HM756656 | *Culiseta melanura* | Northeast | New England | Connecticut | New London | 2003 |
| HM756657 | *Culex pipiens* | Northeast | New England | Connecticut | Fairfield | 2003 |
| HM756658 | *Culiseta melanura* | Northeast | New England | Connecticut | New London | 2003 |
| HM756659 | *Culiseta melanura* | Northeast | New England | Connecticut | Middlesex | 2003 |
| HM756660 | *Accipiter cooperii* | Northeast | Middle Atlantic | New York | Livingston | 2008 |
| HM756661 | *Corvus brachyrhynchos* | Northeast | Middle Atlantic | New York | Bronx | 2001 |
| HM756662 | *Corvus brachyrhynchos* | Northeast | Middle Atlantic | New York | Albany | 2001 |
| HM756663 | *Corvus brachyrhynchos* | Northeast | Middle Atlantic | New York | Albany | 2001 |
| HM756664 | *Corvus brachyrhynchos* | Northeast | Middle Atlantic | New York | Albany | 2002 |
| HM756665 | *Corvus brachyrhynchos* | Northeast | Middle Atlantic | New York | Dutchess | 2002 |
| HM756666 | *Corvus brachyrhynchos* | Northeast | Middle Atlantic | New York | Saratoga | 2003 |
| HM756667 | *Corvus brachyrhynchos* | Northeast | Middle Atlantic | New York | Onondaga | 2003 |
| HM756668 | *Corvus brachyrhynchos* | Northeast | Middle Atlantic | New York | Columbia | 2003 |
| HM756669 | *Corvus brachyrhynchos* | Northeast | Middle Atlantic | New York | Saratoga | 2003 |
| HM756670 | *Corvus brachyrhynchos* | Northeast | Middle Atlantic | New York | Queens | 2003 |
| HM756671 | *Corvus brachyrhynchos* | Northeast | Middle Atlantic | New York | Cortland | 2004 |
| HM756672 | *Corvus brachyrhynchos* | Northeast | Middle Atlantic | New York | Nassau | 2004 |
| HM756673 | *Corvus brachyrhynchos* | Northeast | Middle Atlantic | New York | Oswego | 2004 |
| HM756675 | *Corvus brachyrhynchos* | Northeast | Middle Atlantic | New York | Monroe | 2005 |
| HM756676 | *Cyanocitta cristata* | Midwest | East North Central | Illinois | Perry | 2003 |
| HM756677 | *Loriidae* | West | Mountain | New Mexico | Bernalillo | 2005 |
| HM756678 | *Corvus brachyrhynchos* | Northeast | Middle Atlantic | New York | Jefferson | 2007 |
| HQ671721 | *Corvus brachyrhynchos* | Northeast | Middle Atlantic | New York | Tompkins | 2008 |
| HQ671722 | *Corvus brachyrhynchos* | Northeast | Middle Atlantic | New York | Jefferson | 2002 |
| HQ671723 | *Corvus brachyrhynchos* | Northeast | Middle Atlantic | New York | Putnam | 2003 |
| HQ671724 | *Corvus brachyrhynchos* | Northeast | Middle Atlantic | New York | Broome | 2005 |
| HQ671725 | *Corvus brachyrhynchos* | Northeast | Middle Atlantic | New York | Lewis | 2005 |
| HQ671726 | *Corvus brachyrhynchos* | Northeast | Middle Atlantic | New York | Putnam | 2005 |
| HQ671727 | *Corvus brachyrhynchos* | Northeast | Middle Atlantic | New York | Orleans | 2006 |
| HQ671728 | *Corvus brachyrhynchos* | Northeast | Middle Atlantic | New York | Richmond | 2006 |
| HQ671729 | *Corvus brachyrhynchos* | Northeast | Middle Atlantic | New York | Suffolk | 2006 |
| HQ671730 | *Corvus brachyrhynchos* | Northeast | Middle Atlantic | New York | Onondaga | 2007 |
| HQ671742 | *Cyanocitta cristata* | Midwest | East North Central | Illinois | Perry | 2002 |
| HQ705660 | *Corvus brachyrhynchos* | Northeast | Middle Atlantic | New York | Orange | 2003 |
| HQ705669 | *Cyanocitta cristata* | Midwest | East North Central | Illinois | Clinton | 2002 |
| JF415914 | *Culex quinquefasciatus* | South | West South Central | Texas | Harris | 2005 |
| JF415915 | *Quiscalus quiscula* | South | West South Central | Texas | Harris | 2006 |
| JF415916 | *Mimus polyglottos* | South | West South Central | Texas | Harris | 2006 |
| JF415917 | *Cyanocitta cristata* | South | West South Central | Texas | Harris | 2007 |
| JF415918 | *Cyanocitta cristata* | South | West South Central | Texas | Harris | 2007 |
| JF415919 | *Aedes albopictus* | South | West South Central | Texas | Harris | 2007 |
| JF415920 | *Cyanocitta cristata* | South | West South Central | Texas | Harris | 2007 |
| JF415921 | *Cyanocitta cristata* | South | West South Central | Texas | Harris | 2008 |
| JF415922 | *Culex quinquefasciatus* | South | West South Central | Texas | Harris | 2009 |
| JF415923 | *Culex quinquefasciatus* | South | West South Central | Texas | Harris | 2009 |
| JF415924 | *Cyanocitta cristata* | South | West South Central | Texas | Harris | 2009 |
| JF415925 | *Aedes albopictus* | South | West South Central | Texas | Harris | 2009 |
| JF415926 | *Culex quinquefasciatus* | South | West South Central | Texas | Harris | 2009 |
| JF415927 | *Aedes albopictus* | South | West South Central | Texas | Harris | 2009 |
| JF415928 | *Culex quinquefasciatus* | South | West South Central | Texas | Harris | 2009 |
| JF415929 | *Cyanocitta cristata* | South | West South Central | Texas | Harris | 2005 |
| JF415930 | *Culex quinquefasciatus* | South | West South Central | Texas | Harris | 2006 |
| JF488094 | *Corvus brachyrhynchos* | Northeast | Middle Atlantic | New York | Dutchess | 2004 |
| JF488095 | *Corvus brachyrhynchos* | Northeast | Middle Atlantic | New York | Albany | 2009 |
| JF488096 | *Corvus brachyrhynchos* | Northeast | Middle Atlantic | New York | Suffolk | 2009 |
| JF488097 | *Corvus brachyrhynchos* | Northeast | Middle Atlantic | New York | Suffolk | 2007 |
| JF703161 | *Culex tarsalis* | West | Pacific | California | Imperial | 2004 |
| JF703162 | *Culex tarsalis* | West | Pacific | California | Riverside | 2003 |
| JF703163 | *Culex tarsalis* | West | Pacific | California | Imperial | 2005 |
| JF703164 | *Culex tarsalis* | West | Pacific | California | Riverside | 2003 |
| JF730042 | *Corvus brachyrhynchos* | Northeast | Middle Atlantic | New York | Niagara | 2007 |
| JF899528 | *Corvus brachyrhynchos* | Northeast | Middle Atlantic | New York | Suffolk | 2004 |
| JN183885 | *Cyanocitta cristata* | Northeast | Middle Atlantic | New York | Orleans | 2008 |
| JN183886 | *Cyanocitta cristata* | Northeast | Middle Atlantic | New York | Niagara | 2008 |
| JN183887 | *Corvus brachyrhynchos* | Northeast | Middle Atlantic | New York | Oswego | 2002 |
| JN183891 | *Cyanocitta cristata* | Midwest | East North Central | Illinois | Perry | 2002 |
| JN367277 | *Corvus brachyrhynchos* | Northeast | Middle Atlantic | New York | Niagara | 2004 |
| JX015515 | *Culex tarsalis* | South | West South Central | Texas | El Paso | 2005 |
| JX015516 | *Culex tarsalis* | South | West South Central | Texas | El Paso | 2007 |
| JX015517 | *Culex tarsalis* | South | West South Central | Texas | El Paso | 2008 |
| JX015519 | *Culex quinquefasciatus* | South | West South Central | Texas | El Paso | 2009 |
| JX015521 | *Culex tarsalis* | South | West South Central | Texas | El Paso | 2009 |
| JX015522 | *Culex tarsalis* | South | West South Central | Texas | El Paso | 2010 |
| JX015523 | *Culex tarsalis* | South | West South Central | Texas | El Paso | 2010 |
| KC736486 | *Culex quinquefasciatus* | South | West South Central | Texas | Montgomery | 2012 |
| KC736487 | *Culex quinquefasciatus* | South | West South Central | Texas | Montgomery | 2012 |
| KC736488 | *Culex quinquefasciatus* | South | West South Central | Texas | Montgomery | 2012 |
| KC736489 | *Culex quinquefasciatus* | South | West South Central | Texas | Montgomery | 2012 |
| KC736490 | *Culex quinquefasciatus* | South | West South Central | Texas | Montgomery | 2012 |
| KC736491 | *Culex quinquefasciatus* | South | West South Central | Texas | Dallas | 2012 |
| KC736492 | *Culex quinquefasciatus* | South | West South Central | Texas | Dallas | 2012 |
| KC736493 | *Culex quinquefasciatus* | South | West South Central | Texas | Dallas | 2012 |
| KC736494 | *Culex quinquefasciatus* | South | West South Central | Texas | Montgomery | 2012 |
| KC736495 | *Culex quinquefasciatus* | South | West South Central | Texas | Dallas | 2012 |
| KC736496 | *Culex quinquefasciatus* | South | West South Central | Texas | Montgomery | 2012 |
| KC736497 | *Culex quinquefasciatus* | South | West South Central | Texas | Montgomery | 2012 |
| KC736498 | *Culex quinquefasciatus* | South | West South Central | Texas | Montgomery | 2012 |
| KC736499 | *Culex quinquefasciatus* | South | West South Central | Texas | Montgomery | 2012 |
| KC736500 | *Culex quinquefasciatus* | South | West South Central | Texas | Dallas | 2012 |
| KC736501 | *Culex quinquefasciatus* | South | West South Central | Texas | Dallas | 2012 |
| KC736502 | *Culex quinquefasciatus* | South | West South Central | Texas | Dallas | 2012 |
| KF704147 | *Culex quinquefasciatus* | West | Mountain | Arizona | Maricopa | 2010 |
| KF704153 | *Culex quinquefasciatus* | West | Mountain | Arizona | Maricopa | 2010 |
| KF704158 | *Culex quinquefasciatus* | West | Mountain | Arizona | Maricopa | 2010 |
| KJ786935 | *Mimus polyglottos* | South | West South Central | Texas | Harris | 2012 |
| KJ786936 | *Cyanocitta cristata* | South | West South Central | Texas | Harris | 2012 |
